# Decision-Making Behaviour Evolution Among Pork Supply and Demand Subjects Under Normalisation of COVID-19 Prevention and Control: A Case Study in China

**DOI:** 10.3389/fpubh.2022.784668

**Published:** 2022-03-17

**Authors:** Li Ma, Yidi Wang, Yun Teng

**Affiliations:** ^1^College of Engineering, Northeast Agricultural University, Harbin, China; ^2^Postdoctoral Research Station of Agriculture and Forestry Economics and Management, Northeast Agricultural University, Harbin, China

**Keywords:** quality and safety of agricultural products, evolutionary game, pork supply and demand, decision-making behaviours, the normalisation of COVID-19 prevention and control

## Abstract

Affected by the coronavirus disease 2019 (COVID-19), there were short-term uncertainties in China's live pig industry supply chain. Due to the insufficient supply of pork, the price of pork rose from 33.21 yuan/kg at the end of 2019 to 37.46 yuan/kg in mid-February and fell to 26.41 yuan/kg in mid-May. To restore pig supply and stabilise prices, China issued relevant policies. Given the current effective control of COVID-19 in China, this paper constructed an evolutionary game model of China's pork supply and demand stakeholders under normalisation of COVID-19 prevention and control, analysed the behavioural strategies of consumers, government, and pig farmers, used MATLAB software for data simulation, and expounded on the evolution path and the characteristic rule of tripartite decision-making behaviours. The results showed that government supervision costs, evaluation of government by consumers and pig farmers, government subsidies to pig farmers and consumers, and the proportion of stakeholder behaviours affected the formation of tripartite relationships. The results provide a useful reference for the government to formulate effective policies, increase pig supply, and stabilise pork prices.

## Introduction

The spread of the coronavirus disease 2019 (COVID-19) caused feed and slaughter companies to underoperate and interprovincial and rural roads to be blocked, which gradually increased the cost of breeding, reduced the number of pig farmers, and severely hurt the pig industry (1). China issued several policies to accelerate the recovery of the live pig market affected by COVID-19. The No. 1 Central Document in 2020 stressed that all localities should strictly implement various policies and measures to support live pig production, step up to break the bottleneck of environmental impact assessment, land use, and credit and rectify the problem of arbitrarily expanding the restricted feeding areas. In addition, the document also allowed pig breeding to use generally cultivated land and cancelled the upper limit of 15 mu of land, which improved the resumption of production in the industry. The No. 1 Central Document in 2021 emphasised that pig farming should accelerate the construction of modern breeding systems, protect the basic production capacity of live pigs, and improve the long-term mechanism for the stable and orderly development of the pig industry. With the support of China, pig production capacity is gradually restored. By the end of 2020, the number of live pig stocks and breeding sow stocks increased by 31 and 35.1%, respectively, compared with the end of last year. The output of pork in 2020 was 41.13 million tonnes, which was 3.3% lower than the output of pork in 2019. Whether the restoration of pork supply under normalisation of COVID-19 prevention and control will lead to a decline in pork prices and breeding income has become the most concerning topic for consumers and pig farmers (2).

Pigs are the main source of meat for humans. A moderate amount of pork intake can maintain balanced nutrition for humans. Regarding the role of pork in human nutritional balance (3–5), selenium plays an important role in human health. Fajt et al. (6) collected 135 pork samples from the Czech Republic and analysed the selenium content. The research showed that pork had a significant contribution to the selenium intake of the population of the Czech Republic. An et al. (7) proposed that pork intake, especially fresh/lean pork intake, provided the protein and other essential micronutrients needed by the elderly every day and may have the potential to prevent functional limitations due to nutritional deficiencies. Nong et al. (8) pointed out that pork was the most important source of animal protein for Chinese consumers. The content and composition of fatty acids in pork were closely related to the occurrence and development of metabolic diseases such as cardiovascular disease, obesity, and diabetes.

The ancient Chinese believed that “only pigs can be regarded as family property.” The public has paid close attention to pork supply-demand and pork prices. There has been much research on pork supply and prices (9–11), one of which is the study of Ryu et al. (12) which found that the amount of pork forecasted using structured and unstructured data and five prediction algorithms were more accurate. Zhang and Wang (13) found that a recursive model of the pig herd, a monthly estimation model of new piglets and breeding sows, an estimation model of the initial state of the sow, and a related index of models can more accurately predict the supply of pigs. Chuluunsaikhan et al. (14) implied that the combination of deep learning and news topic models improved the accuracy of forecasting Korean pork prices.

Pork supply and demand are affected by many factors. Academia focuses on the decision-making behaviours of multiple parties in pork supply-demand. There has been much research on multiple parties' decision-making in pig supply and demand (15–17). Zhao et al. (18) used the evolutionary game method to explore the decision-making behaviours of pig farmers and local government in the treatment of faecal pollution of pig scale breeding and found that by adjusting the local government's guidance strength, guidance costs, rewards, punishments, and other factors, pig farmers chose to scientifically treat manure and improve pig survival rate. Liu and Wang (19) used the evolutionary game theory to explore the factors influencing the quality and safety behaviours of pig farmers and slaughtering and processing enterprises and proposed suggestions for pig farmers to increase the supply of qualified pork on the market. Ma et al. (20) applied the evolutionary game method to study the impact of the type of pig farmers' human resources on the scale of pig breeding and concluded that contractual scientific pig farmers had the highest degree of the scale.

To stabilise the pork supply, the government has introduced different policies in accordance with national mechanisms. The policies formulated by the state to stabilise the pork supply are various (21–23). Niemi et al. (24) found that the national policy of generally improving the sanitary conditions of pig fattening could reduce the price of pork by up to 5%. Almazan-Figueroa et al. (25) used a non-linear programming model and found that Mexico's rate adjustment would protect domestic producers while harming pork imports and consumers. Song and Cheung (26) applied the time series analysis method to analyse the impact of pork tariff quotas on household consumption. Pork tariff quotas alleviated the shortage of pork caused by foot-and-mouth disease at the end of 2010.

China also issued several policies, such as improving the pig farm policy and the temporary loan discount policy for large-scale pig farms, optimising the scope of subsidies, and implementing all necessary supplements to ensure pork supply. However, some problems that need to be resolved have been exposed during the implementation of policies. Wang (27) pointed out that the support policy for the pig industry was falling into the problem of the “grassroots implementation dilemma.” The government should adhere to both epidemic prevention and pig supply guarantees and strive to ensure the implementation of relevant national policies. Zhang et al. (2) proposed that although the pig industry was guided by policies, the cost of breeding rose significantly. Coupled with the impact of COVID-19, management costs increased, and the income level of pig farmers was still not optimistic. Liu et al. (28) used VEC modelling with exogenous variables related to the policy to analyse the implementation of the pork collection and storage policy and found that the impact of the policy on pork prices was not significant. After quantitative research, Wang and Zhou (29) discovered the unreasonable part of government subsidies for pig farmers. The pig insurance subsidy policy that could best resist the risk of production fluctuation had the lowest correlation with the stable supply of pigs. The average subsidy level of the incentive policy for pigs transferred out of the county was the highest, but the correlation with the stable supply of pigs was not the highest. The starting point of the government's measures was to give full play to the role of macro-control to protect the interests of consumers and farmers in the case of insufficient supply of live pigs and fluctuations in live pig prices. However, existing studies have shown that national policies still need to be improved in terms of subsidy intensity, fit between policies and people's needs, and grassroots implementation. To give full play to the role of government subsidies and support policies, it is essential to explore the decision-making behaviours of consumers, government, and pig farmers. There is a lack of research on consumers' behaviours and effects on pig supply and demand.

In summary, the paper applies evolutionary game theory to the research on pork supply and demand in China under normalisation of COVID-19 prevention and control and expands the logical relationship of “document combing-model construction-stability analysis-numerical simulation analysis-discussion and conclusions,” reveals the main factors that affect government, pig farmers, and consumers' decision-making and the dynamic evolution of the supply-demand relationship, and explores the balanced policy combination that promotes the balance of pork supply and demand. The research intends to provide more systematic decision support for relevant decision-making departments and promote the formation of an orderly, healthy, and stable pork supply-demand relationship.

## Evolutionary Game Model

Evolutionary game theory is a theory that combines game theory analysis and dynamic evolution process analysis. It no longer models humans as superrational players but believes that humans usually achieve game equilibrium through trial and error. The chosen equilibrium is a function of the equilibrium process to reach equilibrium, and history, institutional factors and certain details of the equilibrium process will all have an impact on the choice of multiple equilibriums in the game. Hirshleifer (30) proposed the concept of evolutionary equilibrium. If the trajectory starting from an arbitrarily small neighbourhood of a certain equilibrium point of the dynamic system eventually evolves towards this equilibrium point, the equilibrium point is said to be locally asymptotically stable, and such a dynamic stable equilibrium point is the evolutionary equilibrium. Finding equilibrium stability points can be achieved by establishing multiparty evolutionary game models, constructing multiparty replication dynamic equations, analysing the behavioural strategies of stakeholders, and measuring equilibrium solutions and stability conditions. These research steps have been widely used in multiparty decision-making research (31–35).

Time occupies a very important position in evolutionary game theory. The theory believes that the Nash equilibrium should be reached after multiple games. In the process of evolution, subjects constantly improve their behaviours and imitate successful strategies. The research on multiparty decision-making in China's pork supply market under normalisation of COVID-19 prevention and control is in line with the characteristics of evolutionary game theory. It is difficult for the three parties to reach the optimal solution at one time in the selection process. Using trial and error, the three parties gradually realise an orderly, stable, and healthy supply and demand relationship.

**Figure 1:**
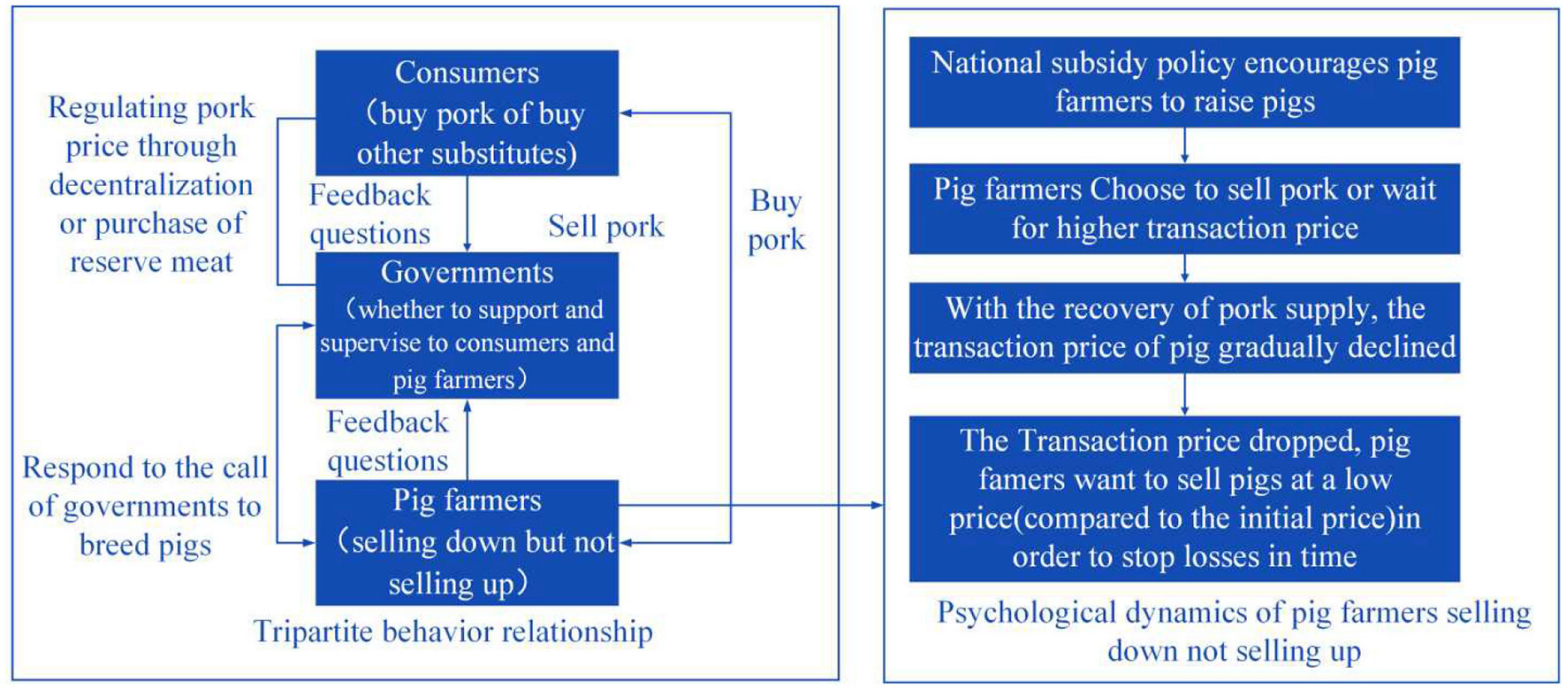
Behaviour relationship among consumers, government, and pig farmers.

Under the normalisation of COVID-19 prevention and control, the supply-demand relationship among consumers, pig farmers, and the government of China is shown in Figure 1: Affected by COVID-19, China's imports of pork decreased. The government introduced subsidy policies to encourage breeding and released reserve meat to adjust the pork supply. Under the normalisation of COVID-19 prevention and control, pig farmers' expectation is to sell pork at a high price to compensate for their losses during the COVID-19 pandemic. When pig farmers wait for a higher transaction price and choose not to sell live pigs, due to the gradual increase in the supply of pork, the transaction price of live pigs falls instead. Facing a decline in transaction prices, pig farmers choose to sell pork to avoid greater losses, resulting in the phenomenon of “sell down but not up” (“sell down but not up” is a conclusion drawn through much actual research, which means that pig farmers sell pork when the transaction price is low, and do not sell pork when the transaction price is high). The reason for the phenomenon is that when facing the high transaction price already existing in the market, pig farmers expect the price to be higher and choose not to trade pigs for the time being (36–38). When prices fall, pig farmers are eager to sell live pigs in order to avoid greater losses. At this time, the actual transaction price is lower.

### Parameter Setting

The parameters of behavioural choice probability are defined as follows: the probability that consumers choose to buy pork is *x*, and the probability that consumers choose not to buy pork is 1−*x*. The probability that the government chooses to supervise and support consumers and pig farmers is *y*, and the probability that government chooses not to supervise and support consumers and pig farmers is 1−*y*. The probability that pig farmers choose to sell products at a high price is *z*, and the probability that pig farmers choose to sell products at a low price is 1−*z*. The value range of *xyz* is as follows: 0 ≤ *x* ≤ 1, 0 ≤ *y* ≤ 1, 0 ≤ *z* ≤ 1.

The parameters of consumers' behaviour strategy are defined as follows: the daily meat demand of consumers is *V*. When supply exceeds demand, low prices stimulate consumers' pork purchase amount to increase to *V*1. When demand exceeds supply, the unit price of pork in the market is *D*1. If the government chooses to supervise and support consumers and pig farmers, it will regulate the pork supply by releasing reserve meat. After the reserved meat is released, the unit price of non-reserve meat decreases from *D*1 to *D*2 (0 < *D*1 < *D*2). Generally, the unit price of reserve meat is 6 yuan lower than the market price, which is set as *D*2−6. The quantity of reserve meat purchased by consumers is *m*, and the quantity of non-reserve meat is *V* − *m* (0 ≤ *m* ≤ *V*). If consumers choose beef and mutton instead of pork, the average unit price of beef and mutton is *D*3. Beef and mutton are short-term substitutes and setting the prices will not be greatly affected in the short term. When the supply exceeds demand, if the government supervises the market, it will regulate the supply to prevent the pork price from being too low. At this time, the unit price of pork is *D*4. If the government does not supervise the market, the unit price of pork is *D*5 (0 < *D*5 < *D*4).

The parameters of the government's behaviour strategy are defined as follows: the government supervision cost to pig farmers and consumers is *J*1, and the cost of the government not supervising consumers and pig farmers is *J*2 (*J*2>*J*1). The evaluation of government by consumers and pig farmers is *S*. The amount of imported pork has decreased, and the state encourages pig farmers to raise live pigs. Pig farmers have learned from the experience of “sell down but not up” and trade at a high price *M*2 in time. At this time, the supply of pork is still gradually recovering and coupled with pig farmers trading at high prices, the unit price of pork in the market will rise. The government regulates the unit price of pork by adjusting the pork supply. The amount of reserve meat is *C*1. If consumers don't buy pork for some reason, the quantity of reserve meat released is *C*2 (*C*2 < *C*1). The government purchases pork as reserve meat in advance to ensure supply and demand, and the unit price of pork purchased by the government is *E*1. Pig farmers are waiting for a high transaction price to compensate for the loss of pigs during the COVID-19 period. However, with the gradual recovery of pork production capacity leading to a decline in the transaction price of live pigs, pig farmers have to sell them at low prices, *M*3. At this time, the unit price of pork in the market decreased. To maintain the balance of pork supply and demand in the future, the government will purchase pork as reserve meat. When consumers buy pork, the government purchases reserve meat at a unit price of *E*2, and the purchase amount is *C*3. When consumers do not purchase pork, the government purchases at a unit price of *E*3 and the purchase amount is *C*4. *B* is government subsidies for pig farmers (including subsidies for improved breeds of pigs, rewards for transferring pigs from large counties, subsidies for standardised scale breeding of pigs, subsidies for breeding sows, and premium subsidies, etc.).

The parameters of pig farmers' behaviour strategy are defined as follows: the transaction price of live pigs before the COVID-19 is *M*1. At present, China's pig production capacity is in the recovery stage. The advocacy of national policies and the shortage of supply in the market will encourage an increasing number of people to breed pigs. After pigs grow up, pig farmers learn from the experience of “sell down but not up” and choose to sell at the transaction price of *M*2. Another part of pig was chosen by farmers to wait for the high price to make up for the loss of breeding pigs during the COVID-19, but when pig farmers witnessed the transaction price declining due to excessive supply, in order to reduce the possible greater loss, pig farmers usually sell pork at the transaction price, *M*3. According to the number of breeds, the national subsidy can be converted into *Q* yuan/pig, the breeding cost is *F* yuan/pig, the breeding and treatment cost of dead pigs is *F*′ yuan/pig, the breeding cost of waiting but selling at a low price is *F*″ yuan/pig. *H* is the cost of transforming into an environmentally friendly pig house, and the number of live pigs is *T*. When pig farmers trade at a high price, consumers purchase pork with the support of government supervision and support, and the hidden benefits brought to pig farmers are *P*.

The above parameters come from the literature and news retrieval. The references for the parameters are shown in Table 1.

**Table 1:** Reference source for model parameters.

**Parameters involved in references**	**References**
*H T D2 D1*	(39)
*H P*	(40)
*B H Q*	(18)
*J1 J2 P Q*	(41)
**F*″ F D2 D1*	(42)
*S T*	(43)
*C2 C1 E2 E1 D5 m1* *V-m1Q*	(44)
*D1 D2 V-m1 m1 D4* *C2 C1 *F*′ E2*	(1)
*E3 E2 C3 C4 C1 E1*	(45) (46)
*M1 M2 M3*	(36) (37)
*D1 D2 D3*	(47) (48)
*V1 V*	(49) (50)

### Game Payoff Matrix

The income matrix among consumers, government, and pig farmers is shown in Table 2. The establishment of the matrix refers to the existing evolutionary game research of stakeholders in the pig industry (18, 20, 51–53).

**Table 2:** The payoff matrix.

**Strategy selection**		**Pig farmers**		
		**Government**	**Sell at a high price z**	**Sell at a low price **(1−*z*)****
Consumers	Buy *x*	Supervise and support *y*	−*m*(*D*2−6)−*D*2(*V*−*m*)*C*1(*D*2−6−*E*1)+*S*−*J*1 *B*+200*T*g*M*2−*FT*g−*F*′*T*(1−g)+*P*−*H*	−*V*1*D*4 *S*−*C*3*E*2−*B*−*J*1 *B*+200*T*g*M*3−*F*″*T*g−*F*′*T*(1−g)−*H* *B*+200*T*g*M*3−*F*″*T*g−*F*′*T*(1−g)−*H*
		No supervise and no support (1−*y*)	−*VD*1 −*S*−*J*2 200*T*g*M*2−*FT*g−*F*′*T*(1−g)−*H*	−*V*1*D*5 −*S*−*J*2 200*T*g*M*3−*F*″*T*g−*F*′*T*(1−g)−*H*
	No buy (1−*x*)	Supervise and support *y*	−*VD*3 *C*2(*D*2−6−*E*1)+*S*−*J*1 *B*+200*T*g*M*2−*FT*g−*F*′*T*(1−g)−*H*	−*VD*3 *S*−*J*1−*B*−*C*4*E*3
		No supervise and no support (1−*y*)	−*VD*3 −*S*−*J*2 200*T*g*M*2−*FT*g−*F*′*T*(1−g)−*H*	−*VD*3 −*S*−*J*2 200*T*g*M*3−*F*″*T*g−*F*′*T*(1−g)−*H*−*P*

## Evolutionary Game Equilibrium Analysis

### Expectation Function

#### Expected Benefits of Consumers

Suppose the expected benefits to consumers buying pork is *E*_e1_, the expected benefits to consumers not buying pork is *E*_e2_ and the average expected benefits to consumers is *E*_*e*_ = *xE*_*e*1_ + (1 − *x*)*E*_*e*2_:


(1) $$\begin{array}{l} E_{e1}=-yz\left[6\text{m}-D2V\right]+y\left(z-1\right)V1D4+z\left(y-1\right)D1V\\ +\left(1-z\right)\left(\text{y}-1\right)V1D5 \end{array}$$

(2) $$\begin{array}{l} E_{e2}=-yzD3V-y\left(1-z\right)D3V-z\left(1-y\right)D3V\\ -\left(1-y\right)\left(1-z\right)D3V \end{array}$$

#### Expected Benefits of Government

Suppose the expected benefits to government supporting and supervising is *E*_*c*1_, the expected benefits to government not supporting and not supervising is *E*_c2_, and the average expected benefits to the government is *E*_*c*_ = *yE*_*c*1_ + (1 − *y*)*E*_*c*2_


(3) $$\begin{array}{l} \begin{array}{c} E_{c1}=xz\left[S-J1+C1\left(D2-6-E1\right)-B\right]\\ +\text{x}\left(1-z\right)\left[S-J1-C3E2-B\right]+\\ \left(1-x\right)z\left[S-J1+C2\left(D2-6-E1\right)-B\right]\\ +\left(1-x\right)\left(1-z\right)\left(S-J1-C4E2-B\right) \end{array} \end{array}$$

(4) $$\begin{array}{l} E_{c2}=xz\left(-S-J2\right)+x\left(1-z\right)\left(-S-J2\right)\\ +z\left(1-\text{x}\right)\left(-S-J2\right)+\left(1-z\right)\left(1-\text{x}\right)\left(-S-J2\right) \end{array}$$

#### Expected Benefits of Pig Farmers

Suppose that the expected benefits to pig farmers selling products at a high transaction price is *E*_*n*1_, the expected benefits to pig farmers selling products at a low transaction price is *E*_*n*2_, and the average expected benefits to pig farmers is *E*_*n*_ = *zE*_*n*1_ + (1 − *z*)*E*_*n*2_


(5) $$\begin{array}{l} E_{n1}=xy[B+200T\text{g}M2-FT\text{g}-F^{\prime }T(1-g)+P-H]\\ +x(1-y)[200TgM2-FTg-F^{\prime }T(1-g)-H]\\ +(1-x)y[B+200TgM2-FTg-F^{\prime }T(1-g)-H]\\ +(1-x)(1-y)[(200TgM3-FTg-F^{\prime }T(1-g)-H-P] \end{array}$$

(6) $$\begin{array}{l} \begin{array}{c} E_{n2}=xy\left[B+200TgM3-FTg-F^{\prime }T\left(1-g\right)-H\right]\\ +x\left(1-y\right)\left[200TgM3-FTg-F^{\prime }T\left(1-g\right)-H\right]\\ +y\left(1-x\right)\left(B+200TgM3-FTg-F^{\prime }T\left(1-g\right)-H\right]\\ +\left(1-x\right)\left(1-y\right)\left[\left(200TgM3-FTg-F^{\prime }T\left(1-g\right)-H-P\right)\right] \end{array} \end{array}$$

### Replicator Dynamic Equation

After calculating consumers' expected benefits above, consumers' replicator dynamic equation is


(7) $$\begin{array}{l} F(x)=\frac{dx}{dt}=x(1-x)(6myz-D2Vyz-V1D4y+yzV1D4\\ -zD1V+zyD1V-V1D5+D3V+V1D5y\\ +V1D5z-yzV1D5) \end{array}$$

After calculating the government's expected benefits above, the government's replicator dynamic equation is


(8) $$\begin{array}{l} F(y)=\frac{dy}{dt}=y(1-y)(C1D2xz-6xzC1-xzC1E1\\ -xC3E2+C3E2xz+zC2D2-6C2z-zC\text{2}E\text{1}-C2D2xz\\ +\text{6}C2xz+E\text{1}xzC2+\text{2}S-J1-C\text{4}E\text{3-}B+xC\text{4}E\text{3}+zC\text{4}E\\ \text{3}-C\text{4}E\text{3}xz+J\text{2}) \end{array}$$

After calculating pig farmers' expected benefits above, pig farmers' replicator dynamic equation is


(9) $$\begin{array}{l} F(z)=\frac{dz}{dt}=z(1-z)[Tg(200M2-200M3+F^{\prime \prime }-F)\\ \;+P(1-x-y+2xy)]. \end{array}$$

### Stability Analysis of Tripartite Strategy Evolution

#### Asymptotic Stability Analysis of Consumers

Calculate the Formula (7) and the results are as follows:


(10) $$\begin{array}{l} x_{1}=0,x_{2}=1,\\ y=\frac{D1Vz+V1D5-D3V-V1D5z}{6mz-D2Vz-V1D4+V1D4z+D1Vz+V1D5-V1D5z} \end{array}$$

(11) $$\begin{array}{l} \frac{dF(x)}{dx}=(1-2x)(6myz-D2Vyz-V1D4y+yzV1D4\\ -zD1V+yzD1V-V1D5+D3V\\ +V1D5y+V1D5z-yzV1D5) \end{array}$$

According to the stability theorem and evolutionary stability strategy of the duplicated dynamic differential equation, when *F*(*x*) = 0 and $\frac{dF\left(x\right)}{dx}<0$, x^*^ is the evolutionary stability strategy.

(1) If $y=\frac{D1Vz+V1D5-D3V-V1D5z}{6mz-D2Vz-V1D4+V1D4z+D1Vz+V1D5-V1D5z}$, *F*(*x*) always equals 0, the results indicate that all levels are in a stable state, and the proportion of consumers' strategy choice will not change over time.

(2) If $y\neq \frac{D1Vz+V1D5-D3V-V1D5z}{6mz-D2Vz-V1D4+V1D4z+D1Vz+V1D5-V1D5z}$, then the $x_{1}^{*}=0,x_{2}^{*}=1$ are the two stable points of *x*. There are two situations:

Situation 1: when $y>\frac{D1Vz+V1D5-D3V-V1D5z}{6mz-D2Vz-V1D4+V1D4z+D1Vz+V1D5-V1D5z}$, if ${\frac{dF\left(x\right)}{dx}|}_{x=1}<0$, ${\frac{dF\left(x\right)}{dx}|}_{x=0}>0$, then $x_{2}^{*}=1$ is the equilibrium point. After measuring factors such as actual needs, the price of substitutes, the price and quantity of reserve meat released by the government, and the unit price of non-reserve pork under government regulation, consumers will buy pork.

Situation 2: when $y<\frac{D1Vz+V1D5-D3V-V1D5z}{6mz-D2Vz-V1D4+V1D4z+D1Vz+V1D5-V1D5z}$, if ${\frac{dF\left(x\right)}{dx}|}_{x=1}>0$, ${\frac{dF\left(x\right)}{dx}|}_{x=0}<0$, then $x_{1}^{*}=0$ is the equilibrium point. Although the government's regulation of pork supply will affect the unit price of pork, the price of pork does not reach the ideal price in the minds of consumers, or consumers themselves have a low willingness to buy pork, consumers will not buy pork at this time.

#### Asymptotic Stability Analysis of Government

Calculate the Formula (8) and the results are as follows:


$$\begin{array}{l} y_{\text{1}}=0,y_{2}=1 \end{array}$$

(12) $$\begin{array}{l} z=\frac{x\text{(}C3E2-C4E3)-2S+J1+C4E3+B-J2}{x(C1D2-6C1-C1E1+C3E2-C2D2+6C2+E1C2-C4E3)+C2(D2-6-E1)+C4E3} \end{array}$$

(13) $$\begin{array}{l} \frac{dF(y)}{dy}=(1-2y)(C\text{1}D\text{2}xz-\text{6}xzC\text{1}-xzC\text{1}E\text{1}-xC\text{3}E\text{2}\\ +C\text{3}E\text{2}xz+zC\text{2}D\text{2}-\text{6}C\text{2}z-zC\text{2}E\text{1}\\ -C\text{2}D\text{2}xz+\text{6}C2xz+xzC\text{2}E\text{1}+2S-J1-C4E3-B\\ +xC4E3+zC4E3-C4E3xz+J2) \end{array}$$

According to the stability theorem and evolutionary stability strategy of the duplicated dynamic differential equation, when *F*(*y*) = 0 and $\frac{dF\left(y\right)}{dy}<0$, y^*^ is the evolutionary stability strategy.

(1) If $z=\frac{x\text{(}C3E2-C4E3)-2S+J1+C4E3+B-J2}{x(C1D2-6C1-C1E1+C3E2-C2D2+6C2+E1C2-C4E3)+C2(D2-6-E1)+C4E3}$, *F*(*y*) always equals 0, the results indicate that all levels are in a stable state, and the proportion of the government's strategy choice will not change over time.

(2) If $z\neq \frac{x\text{(}C3E2-C4E3)-2S+J1+C4E3+B-J2}{x(C1D2-6C1-C1E1+C3E2-C2D2+6C2+E1C2-C4E3)+C2(D2-6-E1)+C4E3}$, then the $y_{1}^{*}=0,y_{2}^{*}=1$ are the two stable points of *y*. There are two situations:

Situation 1: when $z>\frac{x\text{(}C3E2-C4E3)-2S+J1+C4E3+B-J2}{x(C1D2-6C1-C1E1+C3E2-C2D2+6C2+E1C2-C4E3)+C2(D2-6-E1)+C4E3}$, if ${\frac{dF\left(y\right)}{dy}|}_{y=1}<0$, ${\frac{dF\left(y\right)}{dy}|}_{y=0}>0$, then $y_{2}^{\text{*}}=1$ is the equilibrium point. For the government, although the cost of supervision is higher than that of non-supervision, supervision will increase public recognition. If the government pays more attention to the recognition of the public than the cost of supervision, the government will choose to supervise and support pig farmers and consumers at this time.

Situation 2: when $z<\frac{x\text{(}C3E2-C4E3)-2S+J1+C4E3+B-J2}{x(C1D2-6C1-C1E1+C3E2-C2D2+6C2+E1C2-C4E3)+C2(D2-6-E1)+C4E3}$, if ${\frac{dF\left(y\right)}{dy}|}_{y=1}>0$, ${\frac{dF\left(y\right)}{dy}|}_{y=0}<0$, then $y_{1}^{*}=0$ is the equilibrium point. Although the government would like to be recognised by pig farmers and consumers and obtain an improved reputation, the government considers that supervision and support require a lot of time and cost. After measuring the input and output, the government will not support and supervise pig farmers and consumers.

#### Asymptotic Stability Analysis of Pig Farmers

Calculate the Formula (9) and the results are as follows:


(14) $$\begin{array}{l} \;z_{\text{1}}=0,z_{2}=1\\ x=\frac{200Tg(M3-M2)+TgF-TgF^{\prime \prime }-P+P*y}{2yP-P} \end{array}$$

(15) $$\begin{array}{l} \frac{dF(z)}{dz}=(1-2z)(200TgM2-200TgM3\\ +TgF^{\prime \prime }-TgF+2xyP+P-Px-Py) \end{array}$$

According to the stability theorem and evolutionary stability strategy of the duplicated dynamic differential equation, when *F*(*z*) = 0 and $\frac{dF\left(z\right)}{dz}<0$, z^*^ is the evolutionary stability strategy.

(1) If $x=\frac{200Tg\left(M3-M2\right)+TgF-TgF^{\prime \prime }-P+P*y}{2yP-P}$, *F*(*z*) always equals 0, it indicates that all levels are in a stable state, and the proportion of pig farmers' strategy choice will not change over time.

(2) If $x\neq \frac{200Tg\left(M3-M2\right)+TgF-TgF^{\prime \prime }-P+P*y}{2yP-P}$, then the $z_{1}^{*}=0$ $z_{2}^{*}=1$ are the two stable points of *z*. There are two situations:

Situation 1: when $x>\frac{200Tg\left(M3-M2\right)+TgF-TgF^{\prime \prime }-P+P*y}{2yP-P}$, if ${\frac{dF\left(z\right)}{dz}|}_{z=1}<0$, ${\frac{dF\left(z\right)}{dz}|}_{z=0}>0$, then $z_{2}^{*}=1$ is the equilibrium point. Pig farmers learned from the experience of “sell down but not up” and they regard the price was reasonable. At the time, pig farmers will choose to sell pork now.

Situation 2: when $x<\frac{200Tg\left(M3-M2\right)+TgF-TgF^{\prime \prime }-P+P*y}{2yP-P}$, if ${\frac{dF\left(z\right)}{dz}|}_{z=1}>0$, ${\frac{dF\left(z\right)}{dz}|}_{z=0}<0$, then $z_{1}^{*}=0$ is the equilibrium point. To compensate for the losses suffered during the COVID-19, pig farmers hope to wait for a higher transaction price. However, as the production capacity of live pigs is restored, the transaction price of live pigs will fall. Facing the downward trend of transaction prices, pig farmers choose to sell live pigs in order to avoid possible greater losses.

### Model Stability Analysis

According to the concept of *Hirshleifer*, the Jacobian matrix is used to qualitatively analyze the local stability of the above equilibrium points, and the analysis is as follows:


(16) $$\begin{array}{l} J=\left[\begin{array}{ccc} \frac{\partial F\left(x\right)}{\partial x} & \frac{\partial F\left(x\right)}{\partial y} & \frac{\partial F\left(x\right)}{\partial z}\\ \frac{\partial F\left(y\right)}{\partial x} & \frac{\partial F\left(y\right)}{\partial y} & \frac{\partial F\left(y\right)}{\partial z}\\ \frac{\partial F\left(z\right)}{\partial x} & \frac{\partial F\left(z\right)}{\partial y} & \frac{\partial F\left(z\right)}{\partial z} \end{array}\right] \end{array}$$

(17) $$\begin{array}{l} \begin{array}{l} DetJ=J_{11}(J_{22}J_{33}-J_{23}J_{32})+J_{12}(J_{21}J_{33}-J_{23}J_{31})+J_{13}(J_{21}J_{32}\\ -J_{22}J_{31})\\ =J_{11}(J_{22}J_{33}-J_{23}J_{32})+J_{31}(J_{12}J_{23}-J_{13}J_{22}) \end{array} \end{array}$$

Ritzberger and Weibul (54) proposed that only eight boundary points should be considered in the tripartite evolutionary game and the middle point cannot be the equilibrium point. If the stability condition that the three eigenvalues are all negative can be satisfied at the same time, then the point is an asymptotically stable point. The stability analysis of eight boundary points is shown in Table 3.

**Table 3:** Stability analysis of equilibrium solution of a tripartite evolutionary game system.

**Equilibrium solution**	**Eigenvalue symbol**	**Stability**
(0,0,0)	Positive numbers exist	Unstable point
(0,0,1)	Positive numbers exist	Unstable point
(0,1,0)	Positive numbers exist	Unstable point
(0,1,1)	Positive numbers exist	Unstable point
(1,0,0)	Positive numbers exist	Unstable point
(1, 0, and 1)	–, 2*S*+*J*2+*C*1(*D*2−*E*1−6)	Asymptotically
	−*J*1−*B*, –	stable point
(1,1,0)	Positive numbers exist	Unstable point
(1, 1, and 1)	–, −2*S*−*J*2−*C*1(*D*2−*E*1−6)	Asymptotically
	+*J*1+*B*, –	stable point

When −(2*S* + *J*2 + *C*1*D*2 − *C*1*E*1 − 6*C*1 − *J*1 − *B*) < 0, it meets the condition that the signs of the tripartite eigenvalues are all negative. At this time, point (1, 1, and 1) is an asymptotically stable point, and the corresponding tripartite behaviour choice is that consumers purchasing pork, government supervising and supporting consumers and pig farmers, and pig farmers selling products at a high transaction price.

## Simulation Analysis

According to the stability analysis of the equilibrium solution of the tripartite evolutionary game system (1, 1, and 1) and (1, 0, and 1) are asymptotically stable points. Asymptotically stable point (1, 1, and 1) represents the behavioural choices of consumers buying pork, government supervising and supporting consumers and pig farmers, and pig farmers selling products at a high transaction price. Asymptotically stable point (1, 0, and 1) represents the behavioural choice of consumers buying pork, the government not supervising and not supporting consumers and pig farmers, and pig farmers selling products at a high transaction price. Through the above analysis of the stability of the equilibrium solution of the tripartite evolutionary game system, we concluded that government supervision cost, government subsidies to pig farmers and consumers (including the quantity of reserve meat released by the government, the price of non-reserved meat after the release of reserve meat and the price at which government purchase pork as reserve meat), and evaluation of government by consumers and pig farmers will affect the formation of the relationship among the three parties.

Although the actual situation of the pork market varies from place to place, numerical simulation can reveal the law of changes in things to a certain extent (55). The simulation data in the paper comes from relevant news retrieval, field research, and multi-subject interview. After obtaining the data, preprocess the data and set the parameter values in this chapter. The specific data can be seen in Table 4.

**Table 4:** Classifications of data sources.

**Parameter**	**Parameter values**	**Parameter**	**Parameter values**	**Parameter**	**Parameter values**
*V*	60,000	*E*2	13.4	*M*1	10
*V*1	75,000	*C*3	41,000	*M*2	7.5
*D*1	42	*C*4	50,000	*M*3	4.5
*D*2	33	*F*	1,295	*D*3	74
*m*	15,000	*F*′	868	*E*3	13
*D*4	25.5	*P*	100,000	*E*1	12
*V* − *m*	35,000	*T*	50,000	*C*2	9,000
*D*5	15	*C*1	15,000	*F*″	1,600

### Data Simulation of the Cost of Government Supervision

To explore the impact of the cost of government supervision on the tripartite relationship, the cost of government non-supervision is set to 250,000. If the value of the cost of government supervision changes, then the value of *J*1−*J*2 changes. In addition to the data in Table 3, *B* = 10000000 = *S*, *C*1 = 15000, *E*1 = 12, and *D*2 = 33 are set. To guarantee that the simulation results are not affected by the value of (*x, y, z*), the same value of (*x, y, z*) is set.

**Figure 2:**
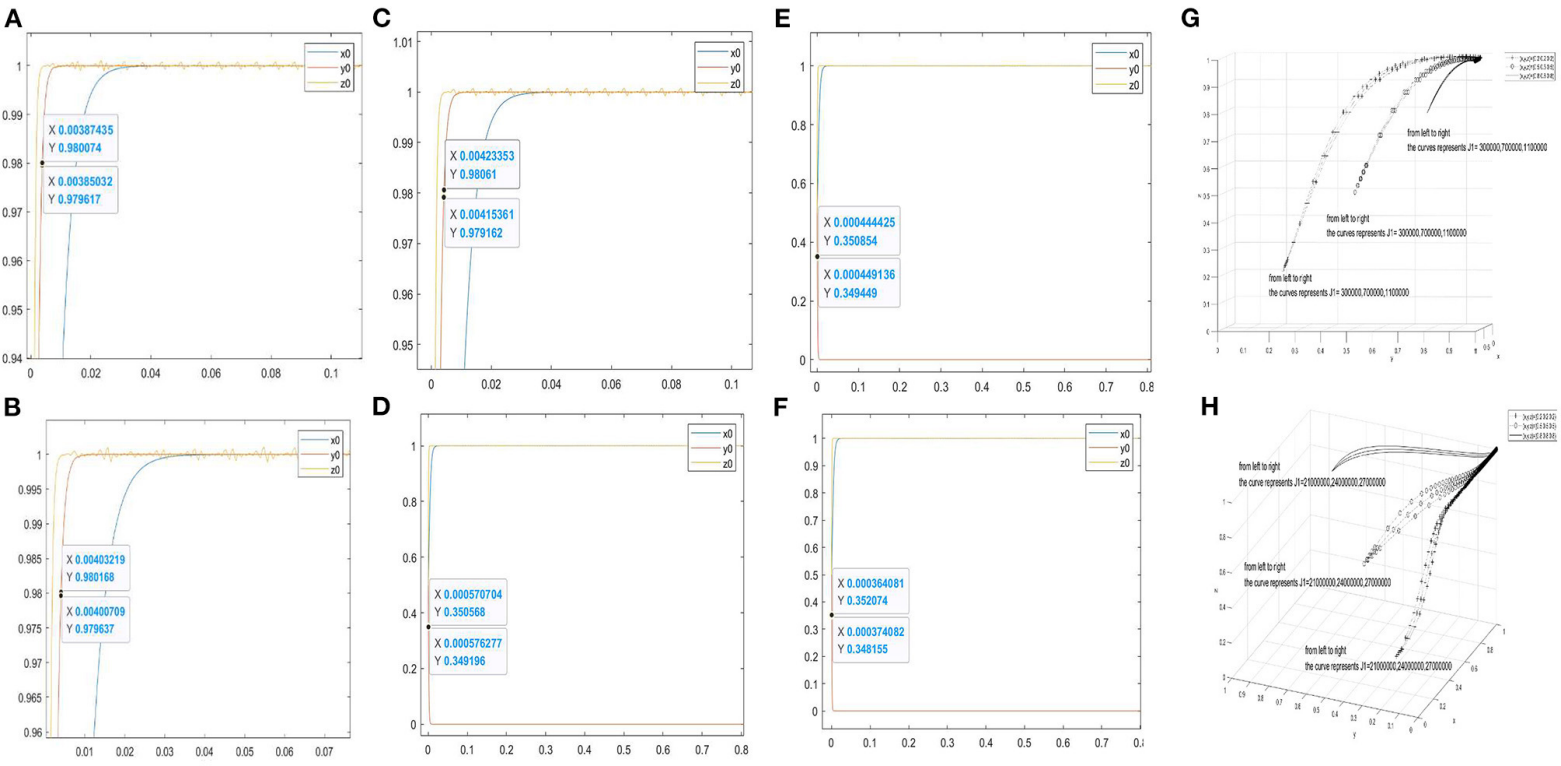
Evolutionary simulation results of the parameter *J*1. **(A–C)** the value range of the abscissa when the ordinate is 0.98 under different factors. **(D–F)** the value range of the abscissa when the ordinate is 0.35 under different factors. **(H,G)** the three-dimensional comparison diagrams under the three-way selection ratio.

Furthermore, Figures 2A–C are the two-dimensional simulation results of *J*1 = 300000, 700000, 1100000. When *y* = 0.98, the corresponding abscissas of the curve *y*_0_ are 0.00385032–0.00387435, 0.00400709–0.00403219, and 0.00415361–0.00423353, respectively. When the stability conditions of the asymptotically stable point (1, 1, and 1) are satisfied, the smaller the value of *J*1 is, the faster the curve *y*_0_ approaches 1. Figures 2D–F are the two-dimensional simulation results of *J*1 = 21000000, 24000000, 27000000. When *y* = 0.35, the corresponding abscissas of the curve *y*_0_ are 0.000570704–0.000576277, 0.00044425–0.00449136, and 0.000364081–0.000374082, respectively. When the stability conditions of the asymptotically stable point (1, 0, and 1) are satisfied, the larger the value of *J*1, the faster the curve *y*_0_ approaches 0.

Moreover, Figures 2G,H are used to observe the effect of *J*1 on the tripartite relationship. Figure 2G shows the three-dimensional simulation results of *J*1 = 300000, 700000, 1100000 under the proportion of stakeholder behaviour choices (0.2, 0.2, and 0.2), (0.5, 0.5, and 0.5), and (0.8, 0.8, and 0.8). Figure 2H shows the simulation results of *J*1 = 21000000, 24000000, 27000000 under the proportion of stakeholder behaviour choices (0.2, 0.2, and 0.2), (0.5, 0.5, and 0.5), and (0.8, 0.8, and 0.8). In Figure 2G, the increase in *J*1 will slow down the curves approach (1, 1, and 1). In Figure 2H, the increase in *J*1 will accelerate the curves approach (1, 0, and 1).

### Data Simulation of Consumers and Pig Farmers' Evaluation of Government

To explore the impact of evaluation of government by consumers and pig farmers on the tripartite relationship, change the value of *S*. In addition to the data in Table 3, *B* = 10000000, *J*2 = 250000, *J*1 = 300000, *C*1 = 15000, *E*1 = 12, *D*2 = 33 are set. To guarantee the simulation results are not affected by the value of (*x, y, z*), the same value of (*x, y, z*) is set.

**Figure 3:**
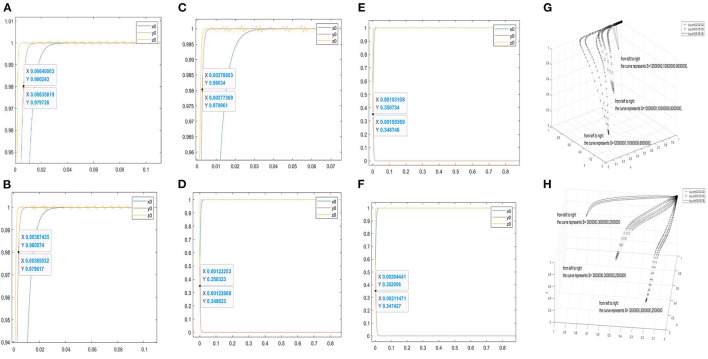
Evolutionary simulation results of the parameter *S*. **(A–C)** the value range of the abscissa when the ordinate is 0.98 under different factors. **(D–F)** the value range of the abscissa when the ordinate is 0.35 under different factors. **(H,G)** the three-dimensional comparison diagrams under the three-way selection ratio.

Furthermore, Figures 3A–C are the two-dimensional simulation results of *S* = 8000000, 10000000, 12000000. When*y* = 0.98, the corresponding abscissas of the curve *y*_0_ are 0.00635819–0.00640003, 0.00385032–0.00387435, and 0.00277369–0.00278803, respectively. When the stability conditions of the asymptotically stable point (1, 1, and 1) are satisfied, the larger the value of *S* is, the faster the curve *y*_0_ approaches to 1. Figures 3D–F are the two-dimensional simulation results of *S* = 2500000, 3000000, 3500000. When *y* = 0.35, the corresponding abscissas of the curve *y*_0_ are 0.00122253–0.00122699, 0.00153108–0.00155369, and 0.00204441–0.00211471. When the stability conditions of (1, 0, and 1) are satisfied, the larger the value of *S*, the slower the curve *y*_0_ approaches 0.

Moreover, Figures 3G,H are used to observe the effect of *S* on the tripartite relationship. Figure 3G shows the three-dimensional simulation results of *S* = 8000000, 10000000, 12000000 under the proportion of stakeholder behaviour choices (0.2, 0.2, and 0.2), (0.5, 0.5, and 0.5), and (0.8, 0.8, and 0.8). Figure 3H shows the three-dimensional simulation results of *S* = 2500000, 3000000, 3500000 under the proportion of stakeholder behaviour choices (0.2, 0.2, and 0.2), (0.5, 0.5, and 0.5), and (0.8, 0.8, and 0.8). In Figure 3G, the increase in *S* will accelerate the curves approach (1, 1, and 1). In Figure 3H, the increase of *S* will slow down the curves approach (1, 0, and 1).

### Data Simulation of Government Subsidies to Consumers

To explore the impact of government subsidies to consumers on the tripartite relationship, change the value of *C*1, *D*2, *E*1. *J*2 = 250000 *J*1 = 300000 are set. To guarantee that the simulation results are not affected by the value of (*x, y, z*), the same value of (*x, y, z*) is set. In this chapter, *C*1, *D*2, *E*1 are discussed, respectively.

#### Parameter *C*1

**Figure 4:**
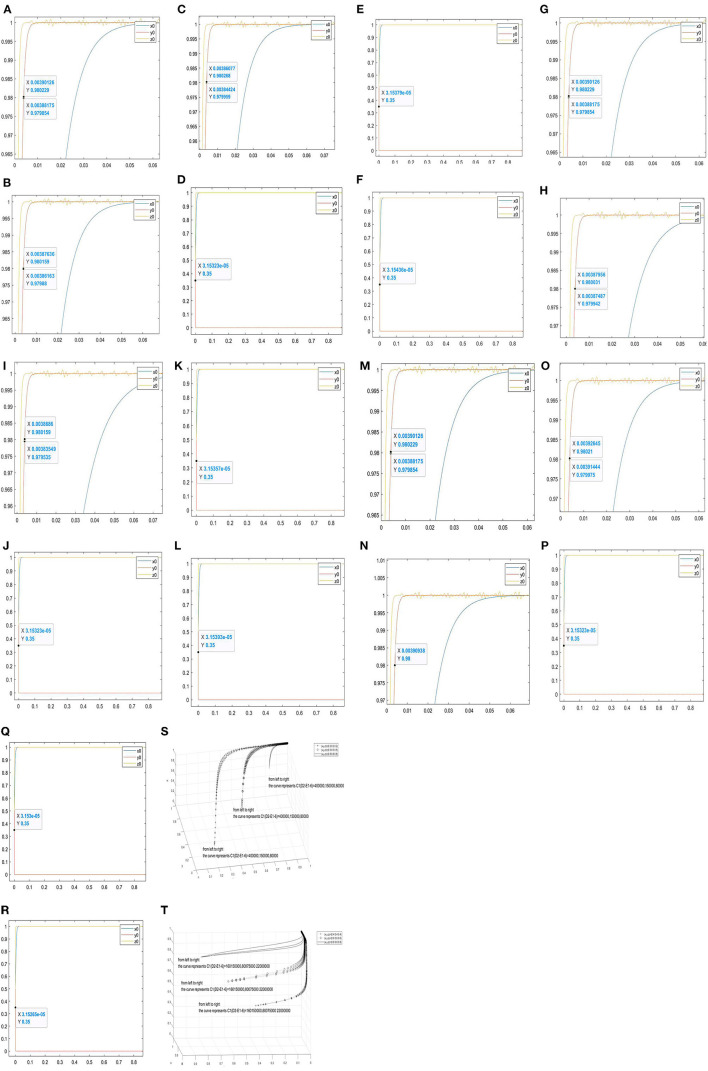
Evolutionary simulation results of the parameter *C*1(*D*2−*E*1−6). **(A–R)** the value range of the abscissa when the ordinate is 0.98 under different factors. **(S,T)** the three-dimensional comparison diagrams under the three-way selection ratio.

When *C*1 = 15000, *E*1 = 15, *D*2 = 31, *C*1(*D*2−*E*1−6) = 75000, the evolutionary results are shown in Figure 4A; when *C*1 = 25000, *C*1(*D*2−*E*1−6) = 125000, the values of other parameters remain unchanged and the evolutionary results are shown in Figure 4B; when *C*1 increases to 35,000, and *C*1(*D*2−*E*1−6) = 175000, the values of other parameters remain unchanged and the evolutionary results are shown in Figure 4C. When *y* = 0.98, the corresponding abscissas of the curve *y*_0_ are 0.00388175–0.00390126, 0.00386163–0.00387636, and 0.00384424–0.00386077, respectively. It can be seen from the figures that the increase of *C*1 will accelerate the curve *y*_0_ approach 1, and the increase of *C*1(*D*2−*E*1−6) will accelerate curves approach (1, 1, and 1).

When *C*1 = 20000, *E*1 = 12, *D*2 = 32, *C*1(*D*2−*E*1−6) = 75000, *B* = 199282000, *S* = 1000, the evolutionary simulation results as shown in Figure 4D; when*C*1 = 30000, *C*1(*D*2−*E*1−6) = 420000, the values of other parameters remain unchanged and the evolutionary results are shown in Figure 4E; when *C*1 = 40000, *C*1(*D*2−*E*1−6) = 560000, the values of other parameters remain unchanged and the evolutionary results are shown in Figure 4F. When *y* = 0.98, the abscissas of the curve *y*_0_ are 3.15323e-05, 3.15379e-05, and 3.15436e-05, respectively. It can be seen from the figures that the increase of *C*1 will slow down the curve *y*_0_ approach 1, and the increase of *C*1(*D*2−*E*1−6) will slow down curves approach (1, 1, and 1).

#### Parameter *D*2

When *C*1 = 15000, *E*1 = 15, *D*2 = 31, *C*1(*D*2−*E*1−6) = 75000, the evolutionary results as shown in Figure 4G; when *D*2 increases to 35, *C*1(*D*2−*E*1−6) = 135000, the values of other parameters remain unchanged and the evolutionary results are shown in Figure 4H; when *D*2 increases to 39, *C*1(*D*2−*E*1−6) = 195000, the values of other parameters remain unchanged and the evolutionary results are shown in Figure 4I. When *y* = 0.98, the corresponding abscissas of the curve *y*_0_ are 0.00388175–0.00390126, 0.00387487–0.0387956, and 0.00383549–0.0038686, respectively. It can be seen from the figures that the increase of *D*2 will accelerate the government curve to 1, and the increase of *C*1(*D*2−*E*1−6) will accelerate curves approach (1, 1, and 1).

When *C*1 = 20000, *E*1 = 12, *S* = 1000, *C*1(*D*2−*E*1−6) = 280000, *B* = 199282000, *D*2 = 32, the evolutionary results as shown in Figure 4J; when *D*2 = 35, *C*1(*D*2−*E*1−6) = 340000, the values of other parameters remain unchanged and the evolutionary results are shown in Figure 4K; when *D*2 = 38, *C*1(*D*2−*E*1−6) = 400000, the values of other parameters remain unchanged and the evolutionary results are shown in Figure 4L. When *y* = 0.98, the corresponding abscissas of the curve *y*_0_ are 3.15323e-05, 3.15357e-05, and 3.15393e-05. It can be seen from the figures that the increase of *D*2 will accelerate the curve *y*_0_ approaches 1, and the increase of *C*1(*D*2−*E*1−6) will accelerate the curves approach (1, 1, and 1).

#### Parameter *E*1

When *C*1 = 15000, *E*1 = 15, *D*2 = 31, *C*1(*D*2−*E*1−6) = 150000, the values of other parameters remain unchanged and the evolutionary results are shown in Figure 4M; when *E*1 increases to 18, *C*1(*D*2−*E*1−6) = 105000, the values of other parameters remain unchanged and the evolutionary results are shown in Figure 4N; when *E*1 increases to 20, *C*1(*D*2−*E*1−6) = 75000, the values of other parameters remain unchanged and the evolutionary results are shown in Figure 4O. When *y* = 0.98, the corresponding abscissas of the curve *y*_0_ are 0.00388175–0.00390126, 0.00390938, and 0.00391444–0.00392645. It can be seen from the figures that the increase of *E*1 will slow down the curve *y*_0_ approaches 1, and the increase of *C*1(*D*2−*E*1−6) will slow down the curves approach (1, 1, and 1).

When *C*1 = 20000, *E*1 = 12, *S* = 1000, *C*1(*D*2−*E*1−6) = 280000, *B* = 199282000, *D*2 = 32, the evolutionary results are shown in Figure 4P; when *E*1 = 14, *C*1(*D*2−*E*1−6) = 240000, the values of other parameters remain unchanged and the evolutionary results are shown in Figure 4Q; when *E*1 = 17, *C*1(*D*2−*E*1−6) = 180000, the values of other parameters remain unchanged and the evolutionary results are shown in Figure 4R. When *y* = 0.98, the corresponding abscissas of the curve *y*_0_ are 3.15323e-05, 3.153e-05, and 3.15265e-05. It can be seen from the figures that the increase of *E*1 will accelerate the curve *y*_0_ approaches 0, and the increase of *C*1(*D*2−*E*1−6)will accelerate curves approach (1, 0, and 1).

Furthermore, Figures 4S,T are used to observe the effect of *C*1(*D*2−*E*1−6) on the tripartite relationship. In Figure 4S, *B* = *S* = 10000000 are set. Figure 4S shows the simulation results of *C*1(*D*2−*E*1−6) = 400000, 150000, 80000 under the proportion of stakeholder behaviour choices (0.2, 0.2, and 0.2), (0.5, 0.5, and 0.5), and (0.8, 0.8, and 0.8). In Figure 4T, *S* = 1000, *B* = 199282000 are set. Figure 4H shows the simulation results of *C*1(*D*2−*E*1−6) = 160150000, 80075000, 22000000 under the proportion of stakeholder behaviour choices (0.4, 0.4, and 0.4), (0.6, 0.6, and 0.6), and (0.8, 0.8, and 0.8) In Figure 4S, the increase of *C*1(*D*2−*E*1−6) will slow down curves approach (1, 1, and 1). In Figure 4T, the increase of *C*1(*D*2−*E*1−6)will accelerate curves approach (1, 0, and 1).

### Data Simulation of Government Subsidies to Pig Farmers

In order to explore the impact of government subsidies to pig farmers on the tripartite relationship, change the value of government subsidies to consumers. In addition to the data in Table 4, *J*2 = 250000, *J*1 = 300000, *S* = 10000000 are set. In order to guarantee simulation results are not affected by the value of (*x, y, z*), the same value of (*x, y, z*) is set.

**Figure 5:**
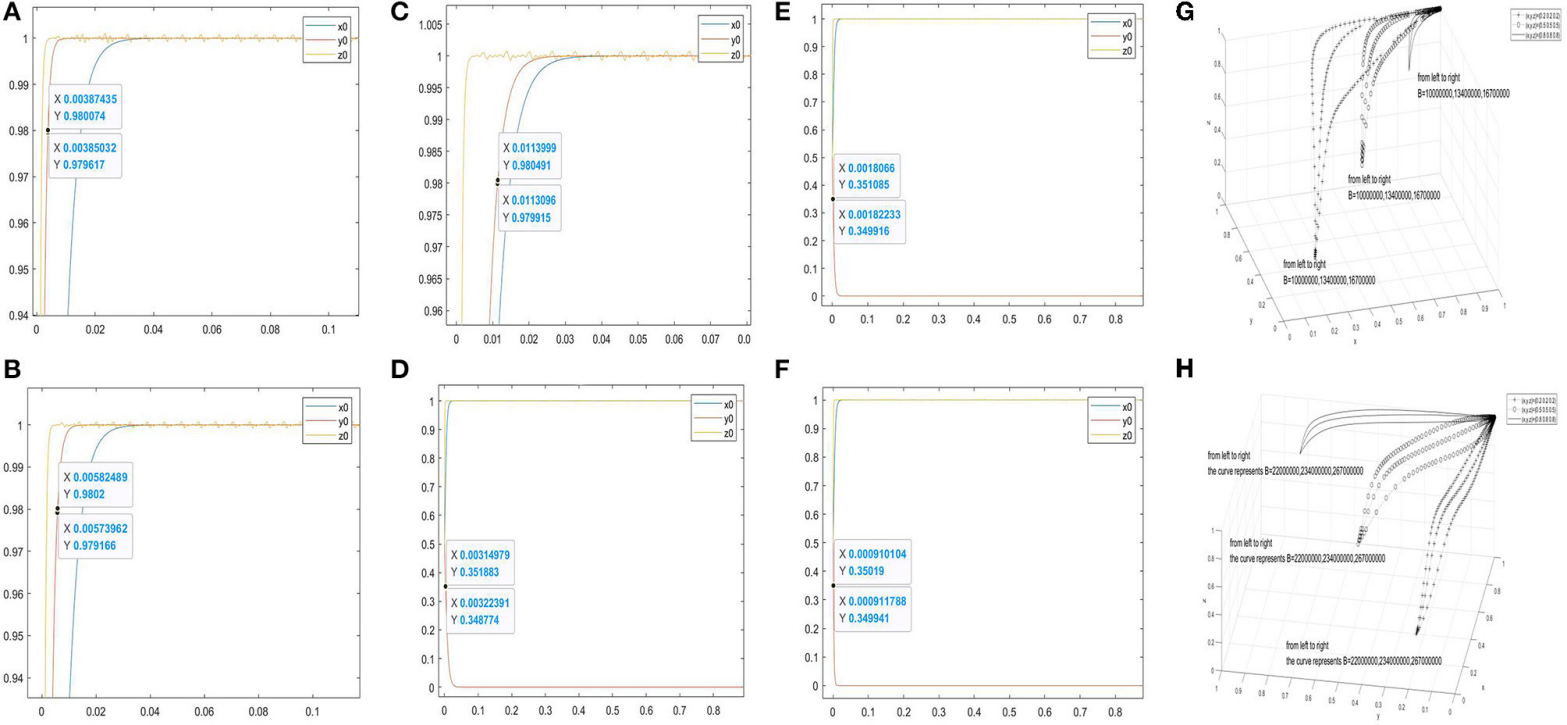
Evolutionary simulation results of the parameter *B*. **(A–C)** the value range of the abscissa when the ordinate is 0.98 under different factors. **(D–F)** the value range of the abscissa when the ordinate is 0.35 under different factors. **(H,G)** the three-dimensional comparison diagrams under the three-way selection ratio.

Moreover, Figures 5A–C are the two-dimensional simulation results of *B* = 10000000, 13400000, 16700000. When *y* = 0.98, the corresponding abscissas of the curve *y*_0_ are 0.00385032–0.00387435, 0.00573962–0.00582489, and 0.0113096–0.0113999. When the stability conditions of (1, 1, and 1) are satisfied, the smaller the value of *B* is, the faster the curve *y*_0_ approaches 1. Figures 5D–F shows the two-dimensional simulation results of *B* = 22000000, 23400000, 26700000. When *y* = 0.35, the corresponding abscissas of the curve *y*_0_ are 0.00317148–0.00324253, 0.0018518–0.00183957, and 0.00914568–0.000918789. When the stability conditions of (1, 0, and 1) are satisfied, the larger the value of *B* is, the faster the curve *y*_0_ approaches 0.

Furthermore, Figures 5G,H are used to observe the effect of *B* on the tripartite relationship. Figure 5G shows the three-dimensional simulation results of *B* = 10000000, 13400000, 16700000 under the proportion of stakeholder behaviour choices (0.2, 0.2, and 0.2), (0.5, 0.5, and 0.5), and (0.8, 0.8, and 0.8). Moreover, Figures 5G,H are used to observe the effect of *B* on the tripartite relationship. Figure 5H shows the three-dimensional simulation results of *B* = 22000000, 23400000, 26700000 under the proportion of stakeholder behaviour choices (0.2, 0.2, and 0.2), (0.5, 0.5, and 0.5), and (0.8, 0.8, and 0.8). In Figure 5G, the increase of *B* will slow down curves approach (1, 1, and 1). In Figure 5H, the increase of *B* will accelerate the curves approach (1, 0, and 1).

### Data Simulation of the Proportion of Tripartite Behaviour Choices

To explore the impact of the proportion of tripartite behaviour choices on tripartite relationships, change the value of the proportion of tripartite behaviour choices (*x, y, z*). In addition to the data in Table 3, *J*2 = 250000, *J*1 = 300000, *C*1 = 15000, *E*1 = 12, *D*2 = 33 are set. In Figures 5A–C, set *B* = *S* = 10000000; In Figures 5D–F, *S* = 1000, *B* = 199282000 are set.

In the first group, the value of (*x, y, z*) is set as (0.2, 0.3, and 0.2), (0.5, 0.6, and 0.5), and (0.8, 0.7, and 0.8). In the second group, the value of (*x, y, z*) is set as (0.4, 0.3, and 0.2), (0.5, 0.8, and 0.5), and (0.8, 0.9, and 0.8). In the third group, the value of (*x, y, z*) is set as (0.4, 0.6, and 0.2), (0.7, 0.6, and 0.9), and (0.8, 0.9, and 0.9). In the fourth group, the value of (*x, y, z*) is set as (0.4, 0.6, and 0.5), (0.7, 0.8, and 0.9), and (0.9, 0.9, and 0.9). The second, third, and fourth groups of data are based on the first group of data to increase the proportion of one, two, and three parties behaviour choices, respectively, to explore the impact of the change in the proportion of tripartite behaviour choices on the three-party supply and demand relationship.

**Figure 6:**
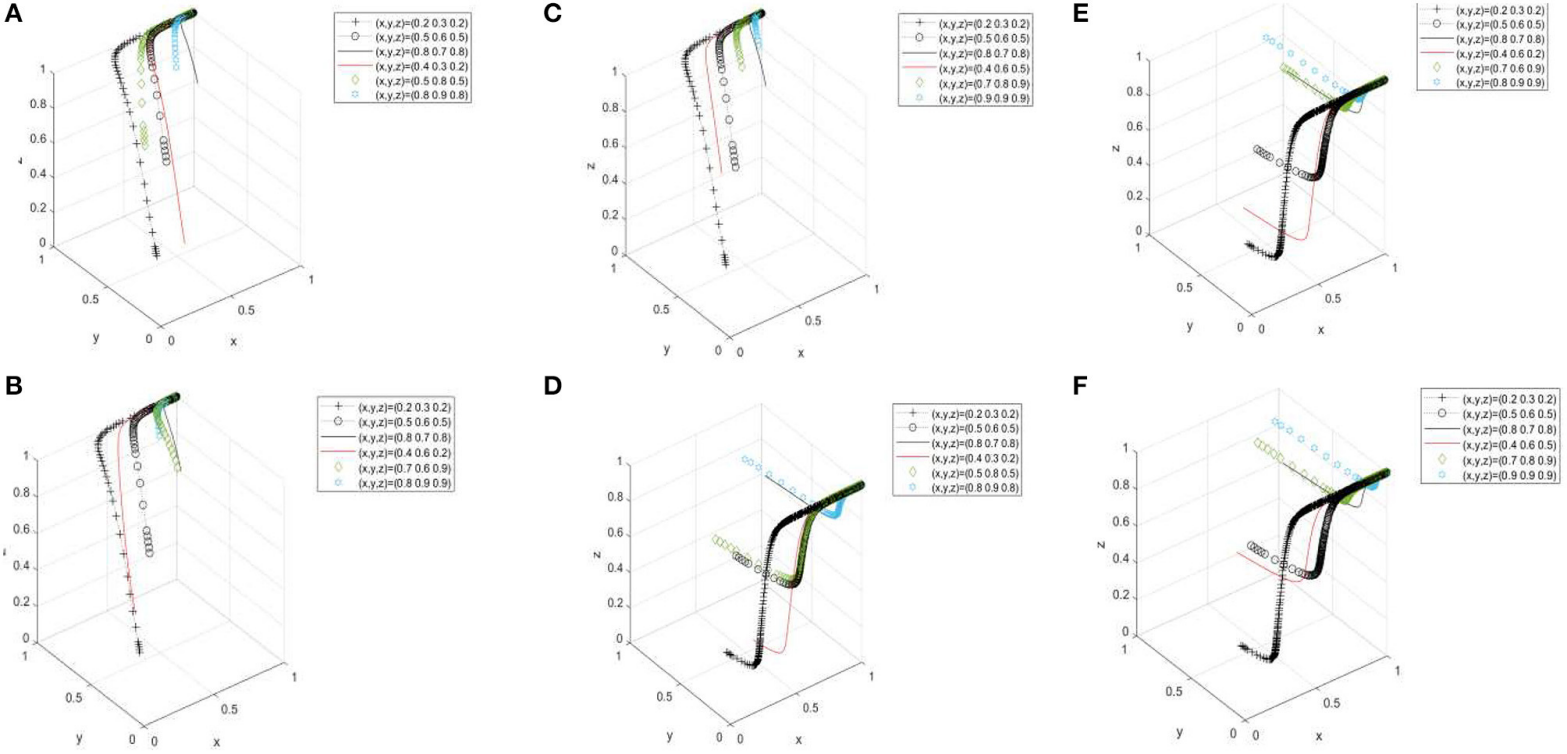
Evolutionary simulation results of the parameter *xyz*. **(A–C)** is a three-dimensional comparison diagram of the three-way behavior selection under the condition of (1, 1, and 1) and the change of (x, y, and z) values. **(D–F)** is a three-dimensional comparison diagram of the three-way behavior selection under the condition of (1, 0, and 1) and the change of (x, y, and z) values.

In Figures 6A–C, under the stability condition of (1, 1, and 1), with the increase of the proportion of one party, two parties, and three parties' behaviour choices, the faster three parties would form the relationship of consumers buying pork-government supervising and supporting consumers and pig farmers- pig farmers selling products at a high transaction price. In Figures 6D–F, under the stability condition of (1, 0, and 1), with the increase of the proportion of one party, two parties, and three parties' behaviour choices, the faster three parties would form the relationship of consumers purchasing pork-government not supervising and not supporting consumers and pig farmers-pig farmers selling products at a high transaction price.

## Discussion And Conclusion

### Discussion

In the results of this study (1, 0, and 1) and (1, 1, and 1) are two asymptotic stability points of the model, and (1, 0, and 1) and (1, 1, and 1) can be converted to each other. (1, 1, and 1) is the ideal pork supply-demand relationship in China. The numerical simulation in the paper proved that the state of (1, 1, and 1) can be realised. Government supervision costs, evaluation of government by consumers and pig farmers, government subsidies to pig farmers and consumers, and the proportion of stakeholder behaviour choices will affect the formation of supply-demand relationships among consumers, the government, and pig farmers in China. The influence of parameter increase on the tripartite relationship is shown in Table 5.

1. When the stability condition of the asymptotic point (1, 1, and 1) is satisfied, the increase in government supervision cost will slow down the speed of the tripartite behaviour curves' approach to (1, 1, and 1). When the stability condition of the asymptotic point (1, 0, and 1) is satisfied, the increase in government supervision cost will accelerate the speed of the tripartite behaviour curves' approach (1, 0, and 1). Strengthening the efficiency of government supervision and reducing the cost of government supervision can accelerate the formation of a stable and orderly supply-demand relationship among the three parties. First, China issued the “Improving the Government's Pork Reserve Regulatory Mechanism and Doing a Good Job in the Work Plan for Maintaining Supply and Stabilising Prices in the Pork Market.” The plan pointed out that to effectively enhance the communication and decision-making efficiency of departments in pork regulation under the normalisation of COVID-19 prevention and control, it's essential to consolidate departmental consultation mechanisms. Second, to improve the authenticity and compliance of government pork regulation, the government should strengthen performance audits and budget planning (56–58). In addition, the government should establish a pork market tracking and monitoring platform to reduce replaceable labour costs (48), standardise the contents, and processes of the pork supervision, and streamline the team.
2. When the stability condition of the asymptotic point (1, 1, and 1) is satisfied, positive evaluation of government by consumers and pig farmers will accelerate the speed of the tripartite behaviour curves' approach to (1, 1, and 1). When the stability condition of the asymptotic point (1, 0, and 1) is satisfied, positive evaluation of government by consumers and pig farmers will slow down the speed of the tripartite behaviour curves' approach to (1, 0, and 1). In the “Improving the Government Pork Reserve Regulation Mechanism and Doing a Good Work Plan for the Pork Market to Guarantee Supply and Stabilise Prices,” China proposed establishing a unified information release platform for the pig industry and pork market. The establishment of the platform is conducive to breaking the barriers of tripartite communication, enabling the government to obtain the timely evaluation from the people, and taking measures to solve problems. The government should enrich the functions of the platform so that consumers can put forward questions and suggestions, pig farmers can feed back their demands on breeding subsidies, and the government can respond to the demands of consumers and pig farmers, giving full play to the role of propaganda position. The “Notice of the General Office of the Ministry of Agriculture and Rural Affairs of the Ministry of Public Security on Combating 'Pig Frying' Behaviours to Ensure the Safety of the Pig Breeding Industry” mentioned that local government should extensively publicise and mobilise to form a strong work momentum, widely publicise a series of measures and achievements in pig market regulation, ensure supply and price stability, epidemic prevention, and strictly investigate “Pig Frying.” The actions will enhance consumer confidence, increase consumer trust in pig safety, and obtain positive comments from consumers and pig farmers.
3. When excessive subsidies to pig farmers make the government unbearable, the increase in government subsidies to pig farmers will slow down the tripartite behaviour curves' approach to (1, 1, and 1), and accelerate the tripartite behaviour curves' approach to (1, 0, and 1). Government subsidies to pig farmers should adhere to the principles of adaptation to local conditions. In terms of subsidies, China issued the “Ministry of Agriculture and Rural Affairs on the issuance of the Three-year Action Plan for Accelerating the Recovery and Development of Live Pig Production,” which involves subsidies for large-scale pig raising, new pig houses, and harmless treatment of sick and dead pigs on farms. Based on existing policies, the government should strictly review the breeding situation, such as the number of live pigs, whether pig farmers have participated in the renovation of environmentally friendly pig houses, and whether new pig farms have been built. In terms of support, China issued the “Implementation Opinions on Supporting Private Enterprises in the Development of Pig Production and Related Industries,” which mentioned that to overcome the special difficulties caused by COVID-19, all localities should increase the support of various refinancing to pig production and related industries. Preferential financing policies and loan interest will encourage pig farmers to continue raising pigs and attract more non-pig farmers to invest in the pig industry.
4. Within a certain range, the increase in government subsidies to consumers will accelerate the tripartite curves' approach to (1, 1, and 1). When the subsidy exceeds the limit, the increase in government subsidies to consumers will accelerate the tripartite curves' approach to (1, 0, and 1). Government subsidies to consumers are mainly through the release and purchase of reserve meat. On the one hand, the government must purchase and release reserve meat in a timely manner. Reserve meat in China is divided into national reserve meat and local reserve meat. National reserve meat is generally released in specific areas when events occur. The local government should actively regulate the market. When the pork price in the local market is excessively abnormal, the local government should actively release reserve meat. Government should buy pork sold in the market when supply exceeds demand, especially when consumers do not want to buy it. At this time, the pork market price is relatively low, and the financial expenditure is lower than the expenditure in the normal reserve. On the other hand, the public needs to increase their recognition of reserve meat. Many consumers consider that reserve meat is not a healthy meat, and they are unwilling to buy reserve meat. Only with the efforts of both parties will the effect of government subsidies on consumers be improved.
5. The increase in the proportion of behaviour of any one or more parties will accelerate the tripartite curves' approach to (1, 1, and 1), and slow down the tripartite curves' approach to (1, 0, and 1). First, to increase consumers' willingness to buy, the government should strengthen the training of pig farmers' hygiene habits, especially the disinfection and cleaning of pig farms. Pig farmers should purchase equipment to ensure hygiene. Second, to improve the government's willingness to supervise, the cost of government supervision should be reduced, manpower and computer technology should be fully integrated, and the monitoring and early warning system of the entire pig industry chain should be improved, so that government can improve and initiate emergency plans in advance and stabilise price across the pig industry chain (59–61). Third, to improve the probability of pig farmers selling pork at a high transaction price, the government should actively communicate with pig farmers, predict the transaction price of pigs, inform pig farmers of the forecast results in a timely manner, and explain in detail the phenomenon of “sell down but not up” and its losses to pig farmers.

**Table 5:** Influence of parameter increase on tripartite relationship.

**Parameter**	**The effect of parameter increasing on the curve approaching (1, 1, and 1)**	**The effect of parameter increasing on the curve approaching (1, 0, and 1)**
Government supervision cost	Slow down	Accelerate
Evaluation of government by consumers and pig farmers	Accelerate	Slow down
Government subsidies to pig farmers	Slow down	Accelerate
Government subsidies to consumers	Accelerate	Accelerate
The proportion of stakeholders' behaviour choices	Accelerate	Slow down

The existing research related to the relationship between pork supply and demand mostly sets the research object as one side of pig farmers or both government and pig farmers, reflects the role of the government through the impact of policies on pig farmers, and proposes countermeasures and suggestions to pig farmers and government, respectively (15–20). There is little research on the role of the government in pork supply and demand. The research considers the relationship between pork supply and demand as the research content and incorporates government, pig farmers, and consumers into the same research framework. The paper refers to the models and parameters of existing literature to construct the relationship between pig farmers and government, innovatively constructs the relationship between consumers and government through field research and document extraction, and fully considers the role of the government for both pig farmers and consumers. Based on the existing literature, the research improves the suggestions to pig farmers and the government and puts forwards innovative suggestions to consumers.

### Conclusions

The research takes China as an example to construct an evolutionary game model of pork supply and demand under the normalisation of COVID-19 prevention and control. The results show that the behavioural decisions of consumers, government, and pig farmers are closely related to the five factors of government supervision costs, government subsidies to consumers and pig farmers, consumers' and pig farmers' evaluations of government, and the probability of the main behaviour choices. The supply-demand relationship can eventually develop in an orderly, stable, and healthy direction. According to the analysis, the following conclusions can be drawn.

First, reducing the cost of government supervision can promote the transformation of the tripartite relationship, from consumers buying pork-government does not supervise and support consumers and pig farmers-pig farmers buying pork at high prices to consumers buying pork-government supervises and supports consumers and pig farmers-pig farmers buying pork at high prices, and finally promoting the formation of orderly pork supply-demand relations among the three parties.

Second, the evaluation of the government's work by consumers and pig farmers is very important to the government's decision-making. Consumers and pig farmers' positive evaluation of the government can encourage the government to take the initiative and earnestly undertake its own supervision and support work. Consumers and farmers' negative evaluation of the government can encourage the government to reflect on the deficiencies in its own work, to assume responsibility, and fulfil its duties in future work.

Third, government subsidies to consumers are mainly through the release and purchase of reserve meat. The purchase and release of reserve meat should grasp the quantity and opportunity. Otherwise, it will lead to a swing between regulatory support and non-regulatory support, which is not conducive to the development of a stable supply-demand relationship between the three parties.

Fourth, government subsidies to pig farmers should follow the principles of strictness, impartiality, and fairness. Excessive subsidies to pig farmers will affect the government's finances and daily operations. Too little subsidies to pig farmers will have a negative effect on pig farmers who are preparing to breed pigs or expand breeding. The government subsidies to pig farmers should be appropriate.

Fifth, a trusting, orderly, and healthy supply relationship relies on the joint efforts of the three parties. If one party's main behaviour choices probability is relatively low, the speed of forming an orderly and healthy pork supply-demand relationship will slow down. Targeted solutions to the concerns of the three parties and attempts to meet the needs of the three parties will promote the formation of a relationship that benefits the three parties.

## Data Availability Statement

The original contributions presented in the study are included in the article/supplementary material, further inquiries can be directed to the corresponding author/s.

## Author Contributions

LM and YT contributed to conception and design of the study and make corrections to the first draft. YDW and LM organised the database. YDW and YT performed the statistical analysis. YDW wrote the first draft of the manuscript. All authors contributed to manuscript revision, read, and approved the submitted version.

## Funding

This research was funded by Heilongjiang philosophy and social sciences research planning project [Grant 21JYD273], Humanities and Social Sciences Foundation of Ministry of Education of China [Grant 18YJC630162], Heilongjiang Province Postdoctoral Science Foundation [Grant LBH-Z17018], Key laboratory Project of Modern Agricultural Equipment Technology in Northern Cold Region [Grant KF18-01], Young Talents of Northeast Agricultural University [Grant 20XG07].

## Conflict of Interest

The authors declare that the research was conducted in the absence of any commercial or financial relationships that could be construed as a potential conflictof interest.

## Publisher's Note

All claims expressed in this article are solely those of the authors and do not necessarily represent those of their affiliated organizations, or those of the publisher, the editors and the reviewers. Any product that may be evaluated in this article, or claim that may be made by its manufacturer, is not guaranteed or endorsed by the publisher.
